# Genomic description of human clinical *Aspergillus fumigatus* isolates, California, 2020

**DOI:** 10.1093/mmy/myad012

**Published:** 2023-01-28

**Authors:** Elizabeth Misas, John Z Deng, Jeremy A W Gold, Lalitha Gade, Natalie S Nunnally, Ourania Georgacopoulos, Meghan Bentz, Elizabeth L Berkow, Anastasia P Litvintseva, Tom M Chiller, Jeffrey D Klausner, Nancy A Chow

**Affiliations:** Mycotic Diseases Branch, Centers for Disease Control and Prevention, Atlanta, Georgia, USA; David Geffen School of Medicine, University of California at Los Angeles, Los Angeles, California, USA; Mycotic Diseases Branch, Centers for Disease Control and Prevention, Atlanta, Georgia, USA; Mycotic Diseases Branch, Centers for Disease Control and Prevention, Atlanta, Georgia, USA; Mycotic Diseases Branch, Centers for Disease Control and Prevention, Atlanta, Georgia, USA; Mycotic Diseases Branch, Centers for Disease Control and Prevention, Atlanta, Georgia, USA; Mycotic Diseases Branch, Centers for Disease Control and Prevention, Atlanta, Georgia, USA; Mycotic Diseases Branch, Centers for Disease Control and Prevention, Atlanta, Georgia, USA; Mycotic Diseases Branch, Centers for Disease Control and Prevention, Atlanta, Georgia, USA; Mycotic Diseases Branch, Centers for Disease Control and Prevention, Atlanta, Georgia, USA; Departments of Population and Public Health Sciences and Medicine, Keck School of Medicine, University of Southern California, Los Angeles, California, USA; Mycotic Diseases Branch, Centers for Disease Control and Prevention, Atlanta, Georgia, USA

**Keywords:** *Aspergillus fumigatus*, whole genome sequencing, genomics, aspergillosis

## Abstract

*Aspergillus fumigatus*, an environmental mold, causes life-threatening infections. Studies on the phylogenetic structure of human clinical *A. fumigatus* isolates are limited. Here, we performed whole genome sequencing of 24 *A. fumigatus* isolates collected from 18 patients in U.S. healthcare facilities in California. Single-nucleotide polymorphism (SNP) differences between patient isolates ranged from 187 to 70 829 SNPs. For five patients with multiple isolates, we calculated the within-host diversities. Three patients had a within-host diversity that ranged from 4 to 10 SNPs and two patients ranged from 2 to 16 977 SNPs. Findings revealed highly diverse *A. fumigatus* strains among patients and two patterns of diversity for isolates that come from the same patient, low and extremely high diversity.

*Aspergillus fumigatus* can cause life-threatening fungal infections (i.e., invasive aspergillosis [IA]) in immunocompromised persons.^1,2^ First-line treatment is triazole antifungal medication, but triazole-resistant IA is increasingly reported.^3^ While most IA cases are sporadic, healthcare-associated outbreaks can occur.^4^

Genomic sequencing can help identify outbreaks and test epidemiologic hypotheses during fungal disease public health investigations^5,6^; however, the utility of this technology for investigating IA outbreaks is unclear because the genetic diversity and phylogenetic structure of *A. fumigatus* in clinical settings have not been widely described using whole genome sequencing (WGS) data. Previously, Pringle et al.^7^ used multi-locus sequence typing for ∼60 isolates and found two groups: *A. fumigatus* ‘fumigatus’ and *A. fumigatus* ‘occultum’. Sewell et al. analyzed short tandem repeats of *A. fumigatus* and identified two major subtypes: Clade A and Clade B. Etienne et al.^8^ used WGS to investigate *A. fumigatus* in the USA and also found the two subtypes Clades A and B.

To use genomic sequencing for IA outbreak investigations, additional studies examining *A. fumigatus* from human clinical specimens are needed. Here, we performed genomic sequencing on *A. fumigatus* isolates from patients in multiple U.S. healthcare settings in Southern California. For 24 *A. fumigatus* isolates representing 18 patients, we performed a single-nucleotide polymorphism (SNP) analysis and described within-host diversity for five patients with multiple isolates.

Additional materials and methods are described in the Supplemental Material. Briefly, the U.S. Centers for Disease Control and Prevention (CDC) received *Aspergillus* spp. isolates and performed species identification, antifungal susceptibility testing (AFST), and WGS (Supplementary Table 1). SNPs were identified from the WGS data and used to generate a phylogenetic tree and pairwise distance comparisons. The phylogenetic tree also included sequences from 10 clinical isolates whose clade determination was previously described in Etienne et al.^8^ (Supplementary Table 2).

Of 42 *Aspergillus* spp. isolates received at CDC with specimen collection dates between February and March 2020, 24 (57%) were identified by matrix-assisted laser desorption/ionization-time of flight^9^ as *A. fumigatus*. For the *A. fumigatus* isolates, specimen source types included sputum (*n* = 18, 75%), bronchoalveolar lavage (BAL) (*n* = 5, 21%), and tracheal aspirate (*n* = 1, 4%). The 24 *A. fumigatus* isolates represented 18 patients (Patient 1–Patient 18); five patients had multiple isolates (Patient 1–Patient 5). Patient 2 had three isolates cultured: two from sputum and one from BAL; the remaining patients’ isolates were cultured from the same specimen source. Patient 5 had two isolates, both recovered from sputum, collected at different facilities. The 18 patients received care in nine healthcare facilities (Facilities A–I) and provide a mixture of inpatient and outpatient services. Four patients (22%) were treated at facility E, six (33%) at facility F; facility G and facility I treated two patients. Two patients were diagnosed with IA, six patients had another diagnosis related to *A. fumigatus* (e.g., allergic bronchopulmonary aspergillosis), and six patients were believed to have *A. fumigatus* colonization; in four patients, the *Aspergillus*-related diagnosis was unknown (Supplementary Table 1).

Of the 24 *A. fumigatus* isolates, AFST was performed for 22 (91%). E-test and broth microdilution revealed that all isolates were susceptible to voriconazole and itraconazole.

Phylogenetic analysis revealed all 24 *A. fumigatus* isolates clustered to Clade B (Supplementary Figure 2). A total of 162 272 SNPs were identified with a median SNP difference of 30 272 SNPs between any two isolates. The minimum pairwise distance observed between patient isolates was 187 SNPs (between Patient 10 sample AFIS5370 and Patient 11 sample AFIS5372), and the maximum was 70 829 SNPs (between Patient 1 sample AFIS5366 and Patient 3 sample AFIS5386). Genetic clustering by facility was not observed (Fig. 1A); median number of SNP differences within facilities (27 492 SNPs) was greater than the median across facilities (19 141 SNPs; *P* < .01). For facilities that served multiple patients, the minimum number of SNPs within a facility was observed at facility E with 6483 SNPs (between Patient 12 and Patient 17), and a maximum number of SNPs was observed at facility F with 70 482 SNPs (between Patient 2 and Patient 3). For within-host genetic diversity, two patterns were observed: a low number of SNP differences in three patients (Patient 1, Patient 3, and Patient 4) that ranged from 4 to 10 SNPs and a high number of SNP differences in two patients (Patient 2 and Patient 5) that ranged from 2 to 16 977 SNPs (Fig. 1B). Patient 2 had three isolates cultured; one isolate was genetically highly distinct from the other two.

**Figure 1.**
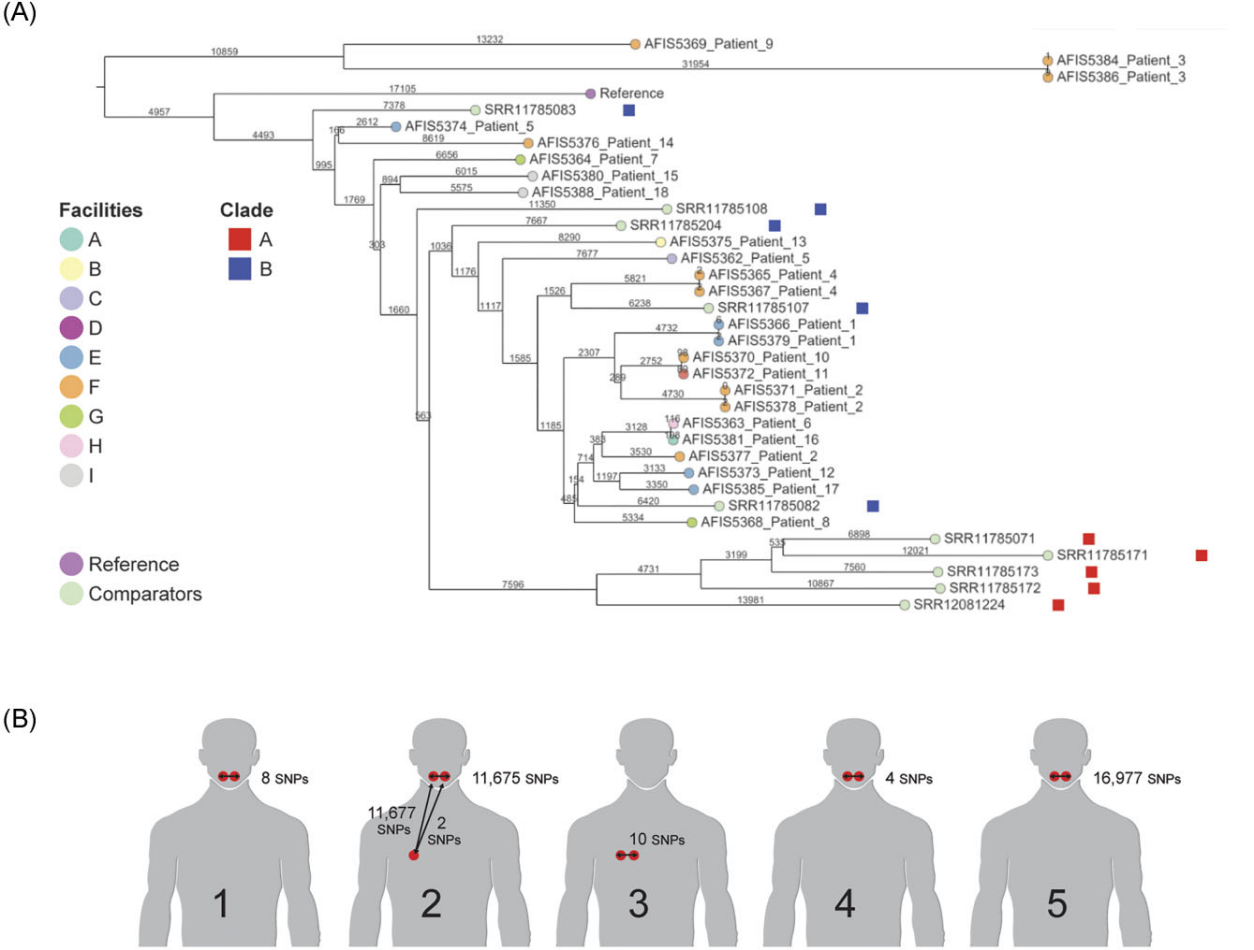
(A) Phylogenetic tree inferred using the neighbor-joining method. The analysis involved 35 nucleotide sequences, 24 isolates from this study, 10 isolates previously described in Etienne et al.,^8^ for clade identification, and the reference sequence, *A. fumigatus* AF293 sequence (GCF_000002655.1), which is labeled in the tree as ‘Reference’. All positions containing gaps and missing data were eliminated. There were a total of 162 272 positions in the final dataset. Evolutionary analyses were conducted in MEGA X and visualized in Microreact. (B) Within-host SNPs differences between patient isolates. Samples from the same patient were collected on different days, except for Patient 1. Two samples from Patient 5 were collected in two different facilities.

Here, we report on antifungal susceptibility, phylogenetic structure, and within-host genetic diversity for 24 *A. fumigatus* isolates from 18 patients. All isolates clustered with Clade B and were susceptible to voriconazole and itraconazole. This is consistent with previous work conducted on U.S. isolates.^8^ Patient isolates did not cluster by facility, and substantial within-host diversity was reported for two patients.

Among the healthcare facilities, hundreds of thousands of SNPs separated patient isolates; the minimum SNP difference between two patient isolates at the same healthcare facility was 6483 SNPs. These results demonstrate that *A. fumigatus* strains across and within the nine healthcare facilities were highly genetically diverse, consistent with similar work looking at *A. fumigatus* from clinical and environmental sources.^10^ Importantly, the presence of *A. fumigatus* is not limited to the healthcare environment, and patients could have acquired *A. fumigatus* outside the healthcare environment. Thus, the diversity presented here may not reflect genetic diversity of *A. fumigatus* found among the healthcare facilities.

We observed a wide range of within-host diversity, three patients (Patients 1, Patient 3, and Patient 4) showing <10 SNP differences between isolates, and two patients (Patient 2 and Patient 5) showing up to >10 000 SNP differences (Fig. 1B). For context, the overall median SNP difference between any two isolates was 30 272 SNPs. Thus, multiple isolates from two of five patients demonstrated substantial within-host diversity, comparable to the genetic diversity between two isolates from any two patients in this study. For Patient 1, Patient 3, and Patient 4, the observed low within-host diversity was likely a result of in-host or in-local environmental genetic drift of a single strain. In contrast, for Patient 2 and Patient 5, the observed high within-host diversity indicated the presence of two different strains. For this, one can consider that in a single exposure event, they inhaled *A. fumigatus* spores comprising two different strains, or it is possible that both Patient 2 and Patient 5 were exposed multiple times to *A. fumigatus*, and that each exposure event involved a distinct strain.

WGS is a powerful tool for testing epidemiologic hypotheses in outbreak investigations, such as linking exposures to contaminated medications or medical devices^5,6^; however, the utility of this method for investigating IA outbreaks has not been demonstrated. In the future, public health officials and healthcare staff may perform WGS on isolates from patients and assess whether genetic relatedness supports an outbreak. The within-host diversity described here highlights that one isolate per patient may not be sufficient to identify linked exposures, and multiple isolates per patient may be needed to establish a baseline SNP difference in the same patient with which to compare.

Our findings have several limitations. Patients’ overlap in time and space was lacking for this study. This affects the finding that patients did not genetically cluster by facility since it may be that the patient isolates that come from the same facility are not related because they did not overlap in time. Finally, isolates included in this study were a result of convenience sampling; therefore, findings observed do not represent the genetic diversity of *A. fumigatus* collected from clinical specimens in healthcare settings in Southern California. Future studies should examine *A. fumigatus* in clinical settings that are experiencing active outbreak investigations. These efforts will continue to advance our understanding of how genomic sequencing can be used to investigate IA outbreaks.

## Supplementary Material

myad012_Supplemental_File
