# Supplementary material for: Genomic description of human clinical Aspergillus fumigatus isolates, California, 2020
Source: Med Mycol. 2023 Jan 28;61(2):myad012. doi: 10.1093/mmy/myad012 (PMC9945844; doi:10.1093/mmy/myad012)
Supplement: myad012_Supplemental_File [file myad012_supplemental_file.zip › mm-2022-0156-File003.docx]

**SUPPLEMENTAL MATERIALS**

*Specimen source*

For the *A. fumigatus* isolates, specimen source types included sputum (n = 18, 75%), bronchoalveolar lavage (BAL) (n = 5, 21%), and tracheal aspirate (n = 1, 4%). The *A. fumigatus* isolates represented 18 patients. For the patient diagnoses, we (JZD and JDK) reviewed medical records to ascertain case-patient diagnoses. The diagnoses were categorized as invasive aspergillosis, colonization, or other based on radiological and clinical findings.

*Antifungal susceptibility testing*

Twenty-two isolates from the collection were screened for elevated minimum inhibitory concentrations (MICs) to itraconazole and voriconazole utilizing Etest strips, according to the manufacturer’s instructions (bioMerieux, Marcy l’Etoile, France)^3^. Susceptibility testing was not performed on two of the isolates due to either insufficient growth or mixed growth, AFIS5385 and AFIS5386 respectively. Isolates with elevated MIC’s (≥2 µg/ml for itraconazole and ≥1 µg/ml for voriconazole) to one or more of the antifungals, were evaluated using the reference broth microdilution method as described in the CLSI document M38^4^. E-test revealed that all isolates were susceptible to voriconazole, and most (91%) were susceptible to itraconazole. The distribution of voriconazole MICs ranged from 0.25–0.5 µg/ml (MIC50: 0.38 µg/ml, MIC90: 0.5 µg/ml), and the distribution of itraconazole MICs ranged from 0.38–2 µg/ml (MIC50: 1.5 µg/ml, MIC90: 1.5 µg/ml). Two isolates displayed elevated minimum inhibitory concentrations to itraconazole but were confirmed to be susceptible to itraconazole via broth microdilution.

*DNA extraction, WGS, and SNP analysis*

DNA was extracted using the DNeasy Blood and Tissue kit (Qiagen, Gaithersburg, MD, USA) according to the manufacturer’s recommendations. Genomic libraries were constructed using NEBNext Ultra DNA Library Prep kit (New England Biolabs, Ipswich, MA, USA) for Illumina and sequenced on Illumina Nova-Seq using NovaSeq 6000SP reagent kit (500 Cycles). Read data has been deposited into the SRA database (BioProject ID PRJNA858199; Supplemental Table 1)

An assembly of *A. fumigatus* AF293 (GCF_000002655.1) comprising nine contigs was used as the reference for read mapping and SNP calling. This reference sequence had a length of 29.4 Mb, GC content of 49.8%, and N50 of 3.95 Mb. For the whole genome SNP analysis, MycoSNP workflow (v0.19) was used [MycoSNP Book chapter; https://git.biotech.cdc.gov/geneflow-workflows/mycosnp/].

The reference genome was masked for repeats using the nucmer command from MUMmer (v4.0)^5^ and Bedtools (v2.29.2)^6^. Post masking, the reference genome was indexed for read alignment using the BWA index command, and for variant calling using Samtools (v 1.10)^7^ faidx and Picards GATK (2.22.9)[http://broadinstitute.github.io/picard/]. Low quality data, trimming, and filtering was performed using FaQCs (v 2.10)^8^. The trimmed reads were used for alignment using the BWA (0.7.17) MEM command^9^. Further, the aligned BAM files from each sample were pr-processed using Samtools and Picard commands and made ready for variant calling. Genome Analysis Toolkit GATK (v 4.1.4.1)^10^ was used for variant calling using the haploid mode. GATK’s VariantFilteration tool was used to filter sites based on the filtering expression “QD < 2.0 || FS > 60.0 || MQ < 40.0”. Using a customized script, genotypes were filtered if the minimum genotype quality was < 50, percentage alternate allele was <0.80 or the depth was < 10.

Variable positions in FASTA format were recovered from the VCF files using a Python script “vcfSnpsToFasta.py” (https://github.com/broadinstitute/broad-fungalgroup/tree/master/scripts/SNPs). For the phylogenetic analysis, 162,272 sites were concatenated. All positions containing gaps or missing data were eliminated. For the resulting sequences, the pairwise distances and Maximum parsimony tree were calculated with the MEGA-X program^11^, and an additional maximum likelihood (ML) phylogenetic tree was constructed with IQtree^12^ (Supplementary Figure 1). Parameters for ML tree were 1,000 bootstraps and the nucleotide substitution transversion model, and unequal base frequency (TVM). For tree visualization, we used the web-based JavaScript application, Microreact^13^.

After determining that the SNP difference values did not have normal distribution using Shapiro-Wilks test (Across facilities W = 0.75351, p-value < 2.2e-16 and Within facilities W = 0.84197, p-value = 7.947e-06 ), a comparison between groups (across-facilities and within-facilities) was estimated using the Mann-Whitney U test for SNP differences for each pair of patients (W = 4067, p-value = 0.002863). The within-host SNPs differences were excluded from the statistic test.

**Supplementary Figure 1**: Maximum likelihood tree of *A. fumigatus* isolates, obtained using IQ-tree version 1.4.4 (best-fit model TVMe + ASC). The data set included a total of 162,272 single nucleotide substitution sites for 35 nucleotide sequences, 24 isolates from this study, 10 isolates previously described in Etienne et. al 2021, for clade identification, and the reference sequence, A. fumigatus AF293 sequence (GCF_000002655.1) which is labeled in the tree as “Reference”. Bootstrap values calculated using 1,000 reiterations are shown.

**Supplemental Table 1:** *A. fumigatus* strains collected from patients in California healthcare facilities. SSR ID correspond to the accession run identification in Sequence Read Archive NCBI

| **Sample ID** | **Patient ID** | **Specimen collection date** | **Specimen source (type)** | **Diagnosis** | **Facility ID** | **SRR ID** |
| --- | --- | --- | --- | --- | --- | --- |
| AFIS5362 | Patient_5 | 2/18/2020 | Sputum | other form of Aspergillosis | C | SRR20117983 |
| AFIS5363 | Patient_6 | 3/7/2020 | Sputum | other form of Aspergillosis | H | SRR20117982 |
| AFIS5364 | Patient_7 | 2/17/2020 | Sputum | suspected colonization | G | SRR20117971 |
| AFIS5365 | Patient_4 | 3/7/2020 | Sputum | suspected colonization | F | SRR20117966 |
| AFIS5366 | Patient_1 | 3/10/2020 | Sputum | unknown | E | SRR20117965 |
| AFIS5367 | Patient_4 | 3/9/2020 | Sputum | suspected colonization | F | SRR20117964 |
| AFIS5368 | Patient_8 | 2/20/2020 | Sputum | suspected colonization | G | SRR20117963 |
| AFIS5369 | Patient_9 | 3/2/2020 | Bronchoalveolar lavage | other form of Aspergillosis | F | SRR20117962 |
| AFIS5370 | Patient_10 | 3/3/2020 | Tracheal aspirate | other form of Aspergillosis | F | SRR20117961 |
| AFIS5371 | Patient_2 | 2/21/2020 | Sputum | Invasive Aspergillosis | F | SRR20117960 |
| AFIS5372 | Patient_11 | 2/25/2020 | Sputum | Invasive Aspergillosis | D | SRR20117981 |
| AFIS5373 | Patient_12 | 2/24/2020 | Sputum | unknown | E | SRR20117980 |
| AFIS5374 | Patient_5 | 2/26/2020 | Sputum | other form of Aspergillosis | E | SRR20117979 |
| AFIS5375 | Patient_13 | 2/28/2020 | Sputum | unknown | B | SRR20117978 |
| AFIS5376 | Patient_14 | 2/28/2020 | Bronchoalveolar lavage | unknown | F | SRR20117977 |
| AFIS5377 | Patient_2 | 2/27/2020 | Sputum | Invasive Aspergillosis | F | SRR20117976 |
| AFIS5378 | Patient_2 | 3/2/2020 | Bronchoalveolar lavage | Invasive Aspergillosis | F | SRR20117975 |
| AFIS5379 | Patient_1 | 3/10/2020 | Sputum | unknown | E | SRR20117974 |
| AFIS5380 | Patient_15 | 3/20/2020 | Sputum | suspected colonization | I | SRR20117973 |
| AFIS5381 | Patient_16 | 3/12/2020 | Sputum | other form of Aspergillosis | A | SRR20117972 |
| AFIS5384 | Patient_3 | 4/27/2020 | Bronchoalveolar lavage | other form of Aspergillosis | F | SRR20117970 |
| AFIS5385 | Patient_17 | 4/27/2020 | Sputum | suspected colonization | E | SRR20117969 |
| AFIS5386 | Patient_3 | 5/1/2020 | Bronchoalveolar lavage | other form of Aspergillosis | F | SRR20117968 |
| AFIS5388 | Patient_18 | 5/18/2020 | Sputum | suspected colonization | I | SRR20117967 |

**Supplemental Table 2:** *A. fumigatus* strains using for comparison. These were sequenced and analyzed by Etienne^1^ et. al 2021.

| **NCBI Accession** | **SRR ID** | **Year Received** | **State** | **Population** |
| --- | --- | --- | --- | --- |
| SAMN15356935 | [SRR12081224](https://trace.ncbi.nlm.nih.gov/Traces/sra/?run=SRR12081224) | 2016 | VA | A |
| SAMN14598560 | [SRR11785171](https://trace.ncbi.nlm.nih.gov/Traces/sra/?run=SRR11785171) | 2017 | CA | A |
| SAMN14598558 | [SRR11785173](https://trace.ncbi.nlm.nih.gov/Traces/sra/?run=SRR11785173) | 2017 | PA | A |
| SAMN14598559 | [SRR11785172](https://trace.ncbi.nlm.nih.gov/Traces/sra/?run=SRR11785172) | 2016 | VA | A |
| SAMN14598491 | [SRR11785071](https://trace.ncbi.nlm.nih.gov/Traces/sra/?run=SRR11785071) | 2016 | PA | A |
| SAMN14598530 | [SRR11785204](https://trace.ncbi.nlm.nih.gov/Traces/sra/?run=SRR11785204) | 2016 | CA | B |
| SAMN14598480 | [SRR11785083](https://trace.ncbi.nlm.nih.gov/Traces/sra/?run=SRR11785083) | 2016 | CA | B |
| SAMN14598457 | [SRR11785108](https://trace.ncbi.nlm.nih.gov/Traces/sra/?run=SRR11785108) | 2015 | CA | B |
| SAMN14598458 | [SRR11785107](https://trace.ncbi.nlm.nih.gov/Traces/sra/?run=SRR11785107) | 2015 | CA | B |
| SAMN14598481 | [SRR11785082](https://trace.ncbi.nlm.nih.gov/Traces/sra/?run=SRR11785082) | 2016 | CA | B |

**SUPPLEMENTAL MATERIALS REFERENCES**

1. Stein M, Tran V, Nichol KA, et al. Evaluation of three MALDI-TOF mass spectrometry libraries for the identification of filamentous fungi in three clinical microbiology laboratories in Manitoba, Canada. *Mycoses*. 2018; 61: 743-753.

2. Etienne KA, Berkow EL, Gade L, et al. Genomic Diversity of Azole-Resistant Aspergillus fumigatus in the United States. *mBio*. 2021; 12: e0180321.

3. Berkow EL, Nunnally NS, Bandea A, Kuykendall R, Beer K, Lockhart SR. Detection of TR34/L98H CYP51A Mutation through Passive Surveillance for Azole-Resistant Aspergillus fumigatus in the United States from 2015 to 2017. *Antimicrob Agents Chemother*. 2018; 62.

4. Institute CaLS. *Reference method for broth dilution antifungal susceptibility testing of filamentous fung*. Clinical and Laboratory Standards Institute, 2017.

5. Delcher AL, Salzberg SL, Phillippy AM. Using MUMmer to identify similar regions in large sequence sets. *Curr Protoc Bioinformatics*. 2003; Chapter 10: Unit 10 13.

6. Quinlan AR, Hall IM. BEDTools: a flexible suite of utilities for comparing genomic features. *Bioinformatics*. 2010; 26: 841-842.

7. Li H, Handsaker B, Wysoker A, et al. The Sequence Alignment/Map format and SAMtools. *Bioinformatics*. 2009; 25: 2078-2079.

8. Lo CC, Chain PS. Rapid evaluation and quality control of next generation sequencing data with FaQCs. *BMC Bioinformatics*. 2014; 15: 366.

9. H L. Aligning sequence reads, clone sequences and assembly contigs with BWA-MEM. 2013.

10. McKenna A, Hanna M, Banks E, et al. The Genome Analysis Toolkit: a MapReduce framework for analyzing next-generation DNA sequencing data. *Genome Res*. 2010; 20: 1297-1303.

11. Kumar S, Stecher G, Li M, Knyaz C, Tamura K. MEGA X: Molecular Evolutionary Genetics Analysis across Computing Platforms. *Mol Biol Evol*. 2018; 35: 1547-1549.

12. Nguyen LT, Schmidt HA, von Haeseler A, Minh BQ. IQ-TREE: a fast and effective stochastic algorithm for estimating maximum-likelihood phylogenies. *Mol Biol Evol*. 2015; 32: 268-274.

13. Argimon S, Abudahab K, Goater RJE, et al. Microreact: visualizing and sharing data for genomic epidemiology and phylogeography. *Microb Genom*. 2016; 2: e000093.
